# HCF-1 promotes cell cycle progression by regulating the expression of CDC42

**DOI:** 10.1038/s41419-020-03094-5

**Published:** 2020-10-23

**Authors:** Pan Xiang, Fei Li, Zhihua Ma, Jiping Yue, Cailing Lu, Yuangang You, Lin Hou, Bin Yin, Boqin Qiang, Pengcheng Shu, Xiaozhong Peng

**Affiliations:** 1 State Key Laboratory of Medical Molecular Biology, Department of Molecular Biology and Biochemistry, Institute of Basic Medical Sciences, Medical Primate Research Center, Neuroscience Center, Chinese Academy of Medical Sciences, School of Basic Medicine Peking Union Medical College, Beijing, China; 2 Institute of Medical Biology, Chinese Academy of Medical Sciences, Peking Union Medical College, Kunming, China

**Keywords:** Chromosome segregation, Chromosome segregation

## Abstract

The eukaryotic cell cycle involves a highly orchestrated series of events in which the cellular genome is replicated during a synthesis (S) phase and each of the two resulting copies are segregated properly during mitosis (M). Host cell factor-1 (HCF-1) is a transcriptional co-regulator that is essential for and has been implicated in basic cellular processes, such as transcriptional regulation and cell cycle progression. Although a series of HCF-1 transcriptional targets have been identified, few functional clues have been provided, especially for chromosome segregation. Our results showed that HCF-1 activated CDC42 expression by binding to the −881 to −575 region upstream of the *CDC42* transcription start site, and the regulation of CDC42 expression by HCF-1 was correlated with cell cycle progression. The overexpression of a spontaneously cycling and constitutively active CDC42 mutant (CDC42F28L) rescued G1 phase delay and multinucleate defects in mitosis upon the loss of HCF-1. Therefore, these results establish that HCF-1 ensures proper cell cycle progression by regulating the expression of CDC42, which indicates a possible mechanism of cell cycle coordination and the regulation mode of typical Rho GTPases.

## Introduction

Successful DNA replication and the accurate segregation of chromosomes are two crucial events during the cell division cycle, which are essential to ensure the extreme fidelity of genome duplication and cell propagation. The progression of these sequential processes is highly regulated, and dysregulation of both aspects contributes to developmental defects or the progression of cancer. Hence, over the past decades, a series of studies in different cell types and genetic models have attempted to reveal the regulatory system that governs the proper transition of defined sequential phases and orchestrates the main events of the cycle.

HCF-1, a highly conserved and abundant chromatin-associated transcriptional cofactor, is known to play a critical role in cell growth and division based on studies of the temperature-sensitive baby hamster kidney (BHK) cell line tsBN67^1–3^. It has been recently established that HCF-1 promotes G1 phase progression and regulates chromosome alignment and segregation. The absence of HCF-1 also results in abnormal M phase^4^. Mutations in HCF-1 as well as the depletion of HCF-1 lead to a multinucleated, primarily binucleated phenotype^2,5^. The regulatory mechanisms of the cell cycle by HCF-1 and its downstream targets remain elusive.

HCF-1 was initially found to be a partner of the transcriptional activator VP16, which is involved in initiating the expression of the herpes simplex virus (HSV) genome^6^ and exists as a family of polypeptides produced by cleavage of the active site of a ~300 kDa precursor protein by O-GlcNAc transferase (OGT)^5,7–9^. Recent studies have demonstrated the DNA binding ability of HCF-1 as a component of different complexes. HCF-1/THAP11/ZNF143 form a complex that binds to ACTACA and TCCCA sub-motifs at target promoters and up- or downregulates gene expression^10,11^. In addition, HCF-1 is a component of the H3K4 methyltransferase SET1/COMPASS complex, which can also bind to the Sin3 histone deacetylase (HDAC) complex that is vital for regulating the cell cycle^12–14^. Several lines of evidence have demonstrated that HCF-1 is recruited to the E2F1 response promoter by MLL family proteins to activate the trimethylation and transcriptional activation of H3K4 and promote the progression of cell cycle G1 to S phase^4,15,16^. Joanna Wysocka and her colleagues reported that HCF-1 can broadly regulate transcription, both positively and negatively, through associations with the Sin3 HDAC and trithorax-related Set1/Ash2l histone methyltransferase (HMT) complexes^17^, eventually leading to the selective modulation of chromatin structure. Genome binding screening of HCF-1 has shown its ability to bind a series of genes involved in different metabolic processes, intergenic sequences and several genes involved in cell cycle regulation under the direction of HCF-1^11^. However, the molecular mechanisms that occur upon the loss of HCF-1, such as multinucleation and chromosome misalignment in particular, have not been fully clarified.

CDC42, a member of the Rho GTPase family, is essential for cell cycle progression through G1 phase and can regulate chromosome alignment and segregation in mammalian cells^18,19^; however, the regulation of CDC42 expression in the cell cycle needs to be further revealed. Here, we find that CDC42 acts downstream of HCF-1. HCF-1 activates CDC42 expression by binding to the −881 to −575 region upstream of the *CDC42* transcription start site to promote G1 progression and mitosis. The overexpression of CDC42 can rescue the decreased expression of Cyclin A after HCF-1 silenced. Together, our findings demonstrate that the expression of CDC42 is regulated by HCF-1 and that it is correlated with cell cycle progression.

## Results

### The expression of CDC42 is reduced in the absence of HCF-1

Various studies have shown that HCF-1 and CDC42 have similar functions in the cell cycle and share almost the same phenotype, for instance, depleting HCF-1 or CDC42 in HeLa cells prevents progression through S phase and causes a cell cycle arrest^4,5,18,19^. HCF-1, a transcription assist activation factor, influences the cell cycle by regulating the transcription of downstream target genes. Therefore, we hypothesized that HCF-1 regulates the expression of CDC42. To determine whether this regulation exists, we analysed the mRNA and protein expression of HCF-1 and CDC42 in HeLa cells and 293ET cells when HCF-1 or CDC42 was depleted by using target plasmids.

**Fig. 1:**
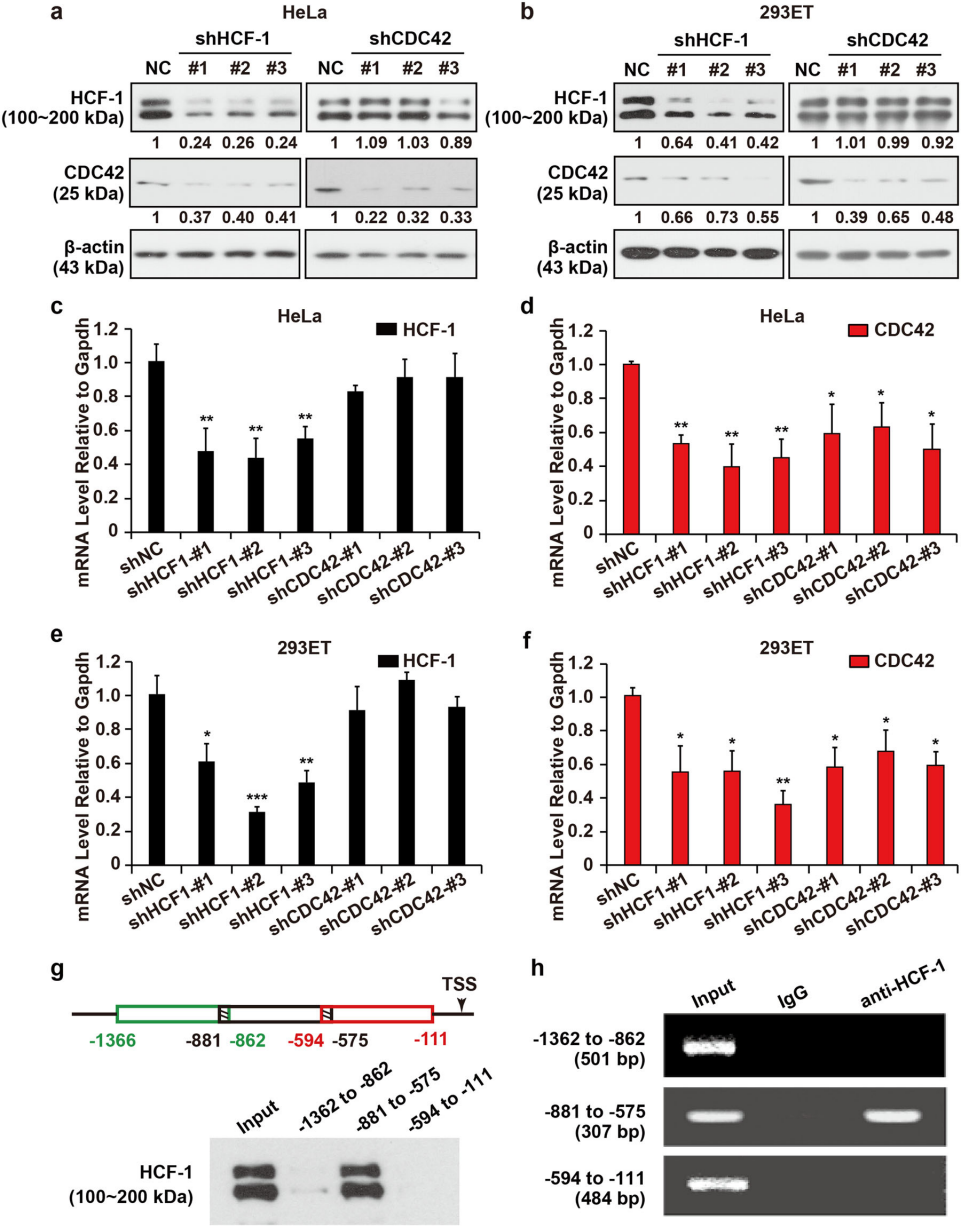
HCF-1 transcriptionally regulates CDC42 expression, and HCF-1 occupies the *CDC42* promoter in vivo. Total protein lysis from HeLa cells and 293ET cells treated with *HCF-1*/*CDC42*-targeting plasmids or nontargeting plasmids (abbreviated NC or shNC) were extracted after 48 h of transfection and then analysed by immunoblotting for HCF-1 and CDC42 expression. β-actin was used as a control. **a** Depletion of HCF-1 or CDC42 led to decreased CDC42 expression in HeLa cells (*n* = 4). **b** Depletion of HCF-1 or CDC42 led to decreased CDC42 expression in 293ET cells. The band intensity values relative to the control were measured with ImageJ software, and the results are shown below the blot (*n* = 4). **c**–**f** The mRNA expression of HCF-1 and CDC42. Depletion of HCF-1 or CDC42 led to decreased CDC42 expression in HeLa cells and 293ET cells (*n* = 3). Results are expressed as mean ± SD. (*p < 0.05, **p < 0.01, ***p < *0.001*. Student’s *t*-test). **g** (Top) A schematic of *CDC42* upstream of the transcription start site is shown. (Bottom) DNA pull-down and immunoblotting assays were used to detect HCF-1 binding in the −1362 to −862, −881 to −575 and −594 to −111 regions of the *CDC42* promoter (*n* = 4). **h** ChIP-PCR analysis of HCF-1 protein on the −1362 to −862, −881 to −575 and −594 to −111 regions of the *CDC42* promoter (*n* = 3).

First, we transfected HeLa cells and 293ET cells with *HCF-1*/*CDC42*-targeting shRNA or a nontargeting control. Cells were collected after 48 h transfection. As shown in Fig. 1a, the treatment of cells with *HCF-1*-targeting shRNA significantly suppressed the expression of endogenous HCF-1 and obviously depleted CDC42 expression, whereas little effect was observed in HeLa cells subjected to *CDC42*-targeting plasmids or control vector. We reached the same conclusion in 293ET cells (Fig. 1b). These effects were also found at mRNA expression level (Fig. 1c–f). Rac1 and RhoA were not altered significantly upon HCF-1 knockdown in Fig. S1a. These results indicate that HCF-1 is involved in the regulation of CDC42 expression. Here, we found that HCF-1 levels remained unchanged upon CDC42 knockdown, which implies that *HCF-1* is more likely to be an upstream gene. Based on the knockdown efficiencies of the *HCF-1*/*CDC42*-targeting plasmids, shHCF-1-#2 (referred to as shHCF-1) and shCDC42-#3 (referred to as shCDC42) were selected for the experiment below.

### HCF-1 binds to the *CDC42* promoter in vivo

To evaluate whether HCF-1 binds to the *CDC42* promoter in vivo, we performed ChIP and DNA pull-down assays using an anti-HCF-1 antibody in HeLa cells. We gota sequence of ~1.5 kb upstream of the *CDC42* gene transcription start site provided in the GenBank database and randomly designed three pairs of primers for amplification of the −1362 to −862, −881 to −575, −594 to −111 segments. As shown in Fig. 1g, a DNA pull-down assay was carried out using HeLa cell extracts. DNA pull-down and immunoblotting assays detected HCF-1 binding to the −1362 to −862, −881 to −575 and −594 to −111 regions of the *CDC42* promoter, while only the −881 to −575 probe obviously recruited HCF-1 for binding. We also used the above primers to identify chromatin fragments obtained by ChIP analysis. The PCR products represented the −881 to −575 region of the *CDC42* promoter but not the −1362 to −862 region or the −594 to −111 region. No band was visible using IgG as a control in the same region (−881 to −575), which was consistent with the DNA pull-down assay (Fig. 1h). These results show that HCF-1 binds to the *CDC42* promoter via the −881 to −575 region.

### Cell cycle-dependent regulation of CDC42 expression by HCF-1

**Fig. 2:**
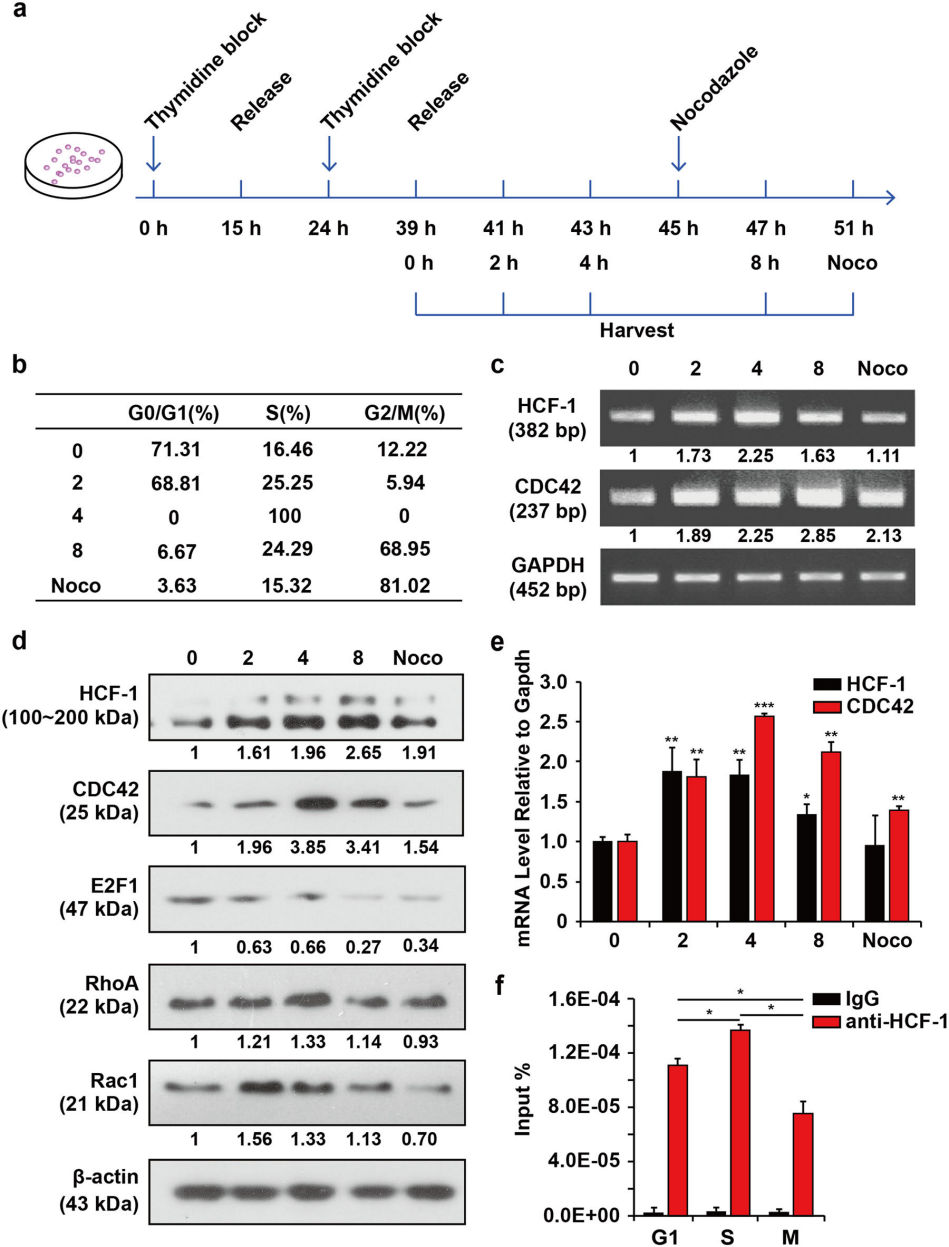
HCF-1 regulates CDC42 expression during different stages of the cell cycle. Flow cytometry analysis of synchronized cell pools. **a** A schematic graph of the synchronization and flow cytometry analysis procedures. The first 15 h thymidine block was performed following 9 h of thymidine release, and cells were harvested at different time points (0, 2, 4, 8 h) after the second 15 h double-thymidine block. One group of synchronized cells was treated with nocodazole 6 h after the second release and harvested after another 6 h for analysis. **b** Synchronized cell pools from different time points were analysed to evaluate their cell cycle distributions (*n* = 3). **c** Cell-cycle-associated changes in the mRNA expression of *HCF-1* and *CDC42* (*n* = 3). The expression levels of HCF-1 and CDC42 in synchronized cell pools were detected by semi-quantitative RT-PCR. The band intensity values relative to the control were measured with ImageJ software, and the number represents the ratio. **d** Cell-cycle-associated changes in the protein levels of HCF-1, CDC42, RhoA and Rac1. The expression levels of HCF-1 and CDC42 in the synchronized cell pool were detected by immunoblotting (*n* = 3). β-actin was used as a loading control, and E2F1 was used as a marker of G1/S phase. The band intensity values relative to the control were measured with ImageJ software, and the results are shown below the blot. **e** qRT-PCR analysis of the mRNA expression of *HCF-1* and *CDC42* at different time points. The comparisons performed were to the respective 0 h controls. Results are expressed as mean ± SD. (*p < 0.05, **p < 0.01, ***p < *0.001*. Student’s *t*-test). **f** ChIP analysis of HCF-1 protein on the −881 to −575 bp region of the *CDC42* promoter throughout the cell cycle (*n* = 3). Absolute quantitative analysis of the content of templates immunoprecipitated with an HCF-1 antibody in different synchronized cell pools (0 h for G1, 4 h for S, and 6 h for the nocodazole-treated cell pool for M phase). Genomic DNA from HeLa cells was serially diluted to generate standard products to establish a standard curve of the relative threshold cycle (Ct). Then, the amount of DNA amount in the ChIP assay was quantified by qRT-PCR according to the standard curve and calculated based on input DNA. Results are expressed as mean ± SD of % input.

Since both HCF-1 and CDC42 are involved in cell cycle regulation and since CDC42 can be regulated by HCF-1, we wondered whether their expression patterns are synchronized. Cell culture and synchronization were performed by the classic double-thymidine block method^20^. As previously reported, cells were enriched in G0/G1 phase upon their release, gradually transitioned to S phase until the peak of S phase almost 4 h after release and then transitioned to G2/M phase at 8 h. Enriched M phase cells were in need of nocodazole treatment. We performed flow cytometry analysis to determine whether our treatment worked. E2F1 is a marker of G1/S phase that was used to demonstrate that the model is correct. As shown in Fig. 2a, HeLa cells were synchronized and collected at different time points after release from G1 arrest and then subjected to flow cytometry analysis (Figs. 2b and S4) and detected by a semi-quantitative RT-PCR assay (Fig. 2c), immunoblotting (Fig. 2d) and quantitative RT-PCR (qRT-PCR) assay (Fig. 2e). The mRNA and protein levels of HCF-1 gradually increased when cells entered S phase (4 h after release from double-thymidine block treatment), peaked during the S phase and declined during mitosis (8 h after release from double-thymidine block treatment and nocodazole treatment). CDC42 mRNA and protein levels gradually accumulated to some extent as cells entered S phase and then underwent G2/M phase accompanied by a reduction. The increase in HCF-1 expression coincided with the upregulation of CDC42 (Fig. 2d), suggesting that HCF-1 is involved in the activation of the *CDC42* promoter during the cell cycle, RT-PCR assay assists this result (Fig. 2c, e). We also examined the amount of the total RhoA, Rac1 during the cell cycle. The protein expression of RhoA and Rac1 has a little variation, both RhoA and Rac1 have a little higher expression in G1 to S phase.

To identify binding differences in different cell phases, we performed ChIP assays of cells in different phases and quantified the amount of DNA by setting up a standard curve of the relative threshold cycle (Ct) of DNA concentration, as shown in Fig. S2. ChIP DNA was then quantified by qRT-PCR according to a standard curve. As shown in Fig. 2f, the amount of HCF-1 on the *CDC42* promoter increased as cells advanced through S phase, and HCF-1 gradually dissociated from the promoter during G2/M phase (8 h after release from double-thymidine block treatment and nocodazole treatment). These results were consistent with the expression pattern of HCF-1 and CDC42.

### CDC42 overexpression suppresses G1 phase arrest upon the loss of HCF-1

**Fig. 3:**
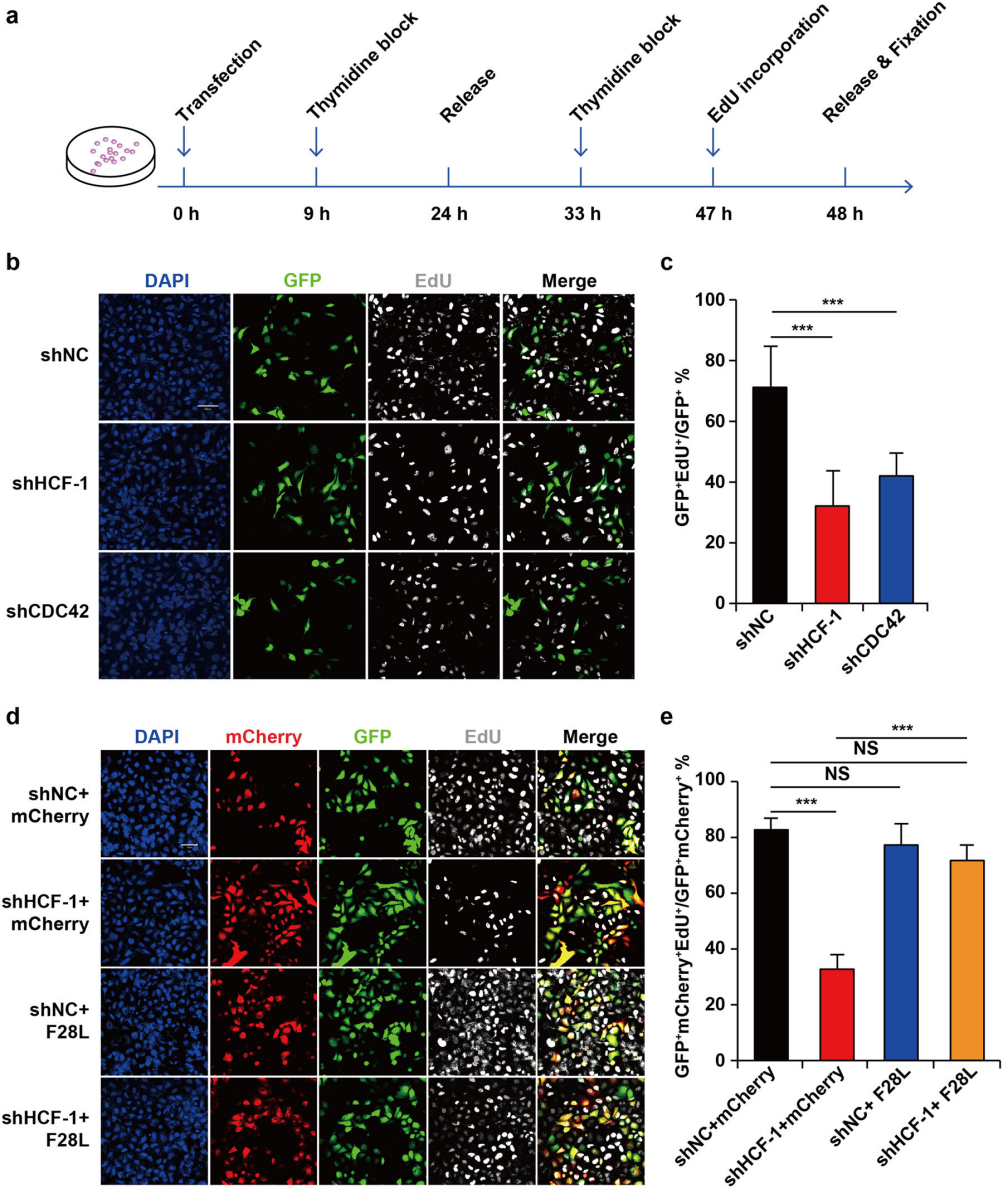
HCF-1 promotes G1 progression by regulating CDC42 expression. HCF-1 and CDC42 depletion induced G1 arrest in HeLa cells. **a** A schematic graph of synchronization and EdU incorporation. The first 15 h thymidine block was performed 9 h after cell transfection, followed by 9 h thymidine release and the second 15 h thymidine block. EdU was added to the culture medium 1 h before the second release and cell fixation. **b** Cells were transfected with shRNA-producing plasmids and Green fluorescent protein (GFP) was expressed in successfully transfected cells. Scale bar: 50 µm. **c** Quantification of EdU incorporation after HCF-1 and CDC42 knockdown. The ratio of EdU incorporation was 70.05, 34.58 and 41.12% in transfected groups, respectively (*n* = 6). **d** Cells were transfected with the shHCF-1 and CDC42F28L vectors. Both GFP and mCherry were expressed in successfully transfected cells. Scale bar: 50 µm. **e** Quantification of EdU incorporation in the co-expression cells. The ratio of EdU incorporation was 81.72, 34.79, 78.37 and 71.45% in transfected groups, respectively (*n* = 8). Results are expressed as mean ± SD. (***p < *0.001*; NS no significant difference. Student’s *t*-test). Replicates and supplemental data showed similar phenotypes and variation trends.

To evaluate whether the regulatory relationship between HCF-1 and CDC42 takes part in G1-S progression, an EdU incorporation assay was performed. During the double-thymidine block procedure, HeLa cells were transfected with a *CDC42*-targeting plasmid, shCDC42, and then incubated with EdU for 1 h to ensure that all cells in which DNA replication occurred were labelled as they passed through S phase. Immunoblotting analysis of CDC42 to evaluate knockdown in HeLa cell extracts revealed effective suppression of endogenous protein levels compared to those in the control cells (Fig. 1a). Cell treatment was shown in Fig. 3a, which was similar to a previous report using Swiss 3T3 fibroblasts expressing the dominant negative form of CDC42^18^, and the protein level of CDC42 was shown in Fig. S1b; ~41.12% of cells treated with shCDC42 were labelled with EdU reagent, whereas 70.05% of control cells showed EdU incorporation (Fig. 3b, c). These results indicate that CDC42 is essential for DNA synthesis in HeLa cells.

*HCF-1* is a broad and key promoter of cell growth and proliferation in mammalian cells. HCF-1 depleted HeLa cells were arrested in the G1 phase (Fig. 3b, c)^5^. Since *CDC42* is the transcriptional target of HCF-1, whether CDC42 overcomes the cell proliferation defect induced by the loss of HCF-1 is verified. It was supposed that a CDC42 molecular switch might spontaneously provide more reliable information on regulation at the molecular level compared to that in the wild-types for detecting the effect of CDC42 in the cell cycle, as the expression of HCF-1 does not change. The overexpression of mCherry fusion protein in HeLa cells was confirmed by immunoblotting (Fig. S3). To examine this hypothesis, HeLa cells were cotransfected with vectors producing HCF-1-targeting shRNA and a spontaneously cycling CDC42 mutant, F28L; this mutant binds GTP in the absence of a guanine nucleotide exchange factor but still hydrolyses GTP with a turnover number identical to that of wild-type CDC42 and is used to maintain full GTPase activity^21^. One-hour EdU incorporation was measured after 48 h of cotransfection as well as double-thymidine block. There was no difference in the number of cells in the control group, *HCF-1* shRNA-treated or *CDC42* shRNA-treated samples, even though a substantial number of cells failed to incorporate EdU. Figure 3b, c shows that cells treated with shHCF-1 failed to be labelled by EdU as a control group, with the incorporation percentage decreasing to 34.58%. In contrast, the co-expression of CDC42F28L in HCF-1-depleted cells rescued the proliferation arrest by more than half (Fig. 3d, e). These results suggest that CDC42 is efficient in rescuing G1 arrest in the absence of HCF-1.

### CDC42 overexpression rescues multinucleation and chromosome misalignment caused by HCF-1 knockdown

**Fig. 4:**
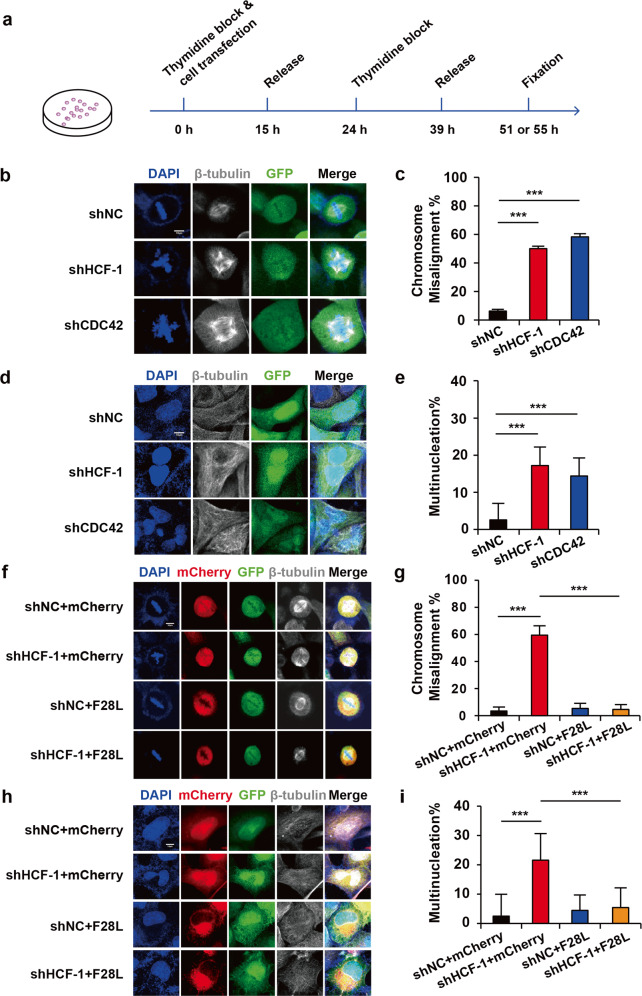
HCF-1 promotes mitosis by regulating CDC42 expression. HCF-1 and CDC42 depletion induced chromosome misalignment and multinucleation in HeLa cells. **a** A schematic graph of synchronization and cell phenotype detection. Cell transfection and the first 15 h thymidine block were performed the same time, followed by 9 h of thymidine release and the second 15 h thymidine block. Cells were collected 12 h after thymidine release for chromosome misalignment detection and 16 h after thymidine release for multinucleate phenotype detection. **b**, **d**, **f**, **h** The quantification is acquired via counting the ratio of the number of mutated cells to the number of normal cells in a confocal resolvable plane in a random field of view. **b**, **d** Cells were transfected with shRNA-producing plasmids. GFP was expressed in successfully transfected cells. Cells were counter-stained with β-tubulin antibody. Scale bar: 10 µm. **c** Quantification of chromosome misalignment after HCF-1 and CDC42 knockdown. The ratio of chromosome misalignment was 6.2, 50 and 58.3% in transfected groups, respectively (*n* = 15). **e** Quantification of multinucleation after HCF-1 and CDC42 knockdown. The ratio of chromosome misalignment was 2.5, 17.2 and 14.3% in transfected groups, respectively (*n* = 10). **f**, **h**) Cells were transfected with shHCF-1 and CDC42F28L vectors. Both GFP and mCherry were expressed in successfully transfected cells. Cells were counter-stained with β-tubulin antibody. Scale bar: 10 µm. **g** Quantification of chromosome misalignment in the co-expression cells. The ratio of chromosome misalignment was 3.4, 59.5, 4.3 and 4.2% in transfected groups, respectively (*n* = 15). **i** Quantification of multinucleation in the co-expression cells. The ratio of chromosome misalignment was 2.5, 21.6, 4.5 and 5.4% in transfected groups, respectively (*n* = 10). Results are expressed as mean ± SD. (***p < *0.001*. Student’s *t*-test). Replicates and supplemental data showed similar phenotypes and variation trends.

The loss of HCF-1 function leads to the multinucleated phenotype of tsBN67 at nonpermissive temperatures in and predominant binucleation defects^2^. Eric Julien and Winship Herr have shown that the HCF-1C subunit is important for mitotic chromosome alignment and segregation^4^. CDC42 is the primary GTPase that functions during mitosis and meiosis^22–24^, and chromosome misalignment and a multinucleated phenotype are induced significantly in HeLa cells by CDC42 depletion^23^. We wondered whether the overexpression of CDC42, as a transcriptional target of HCF-1, rescues the multinucleated defect in the absence of HCF-1. We performed cell transfection along with double-thymidine block, as shown in Fig. 4a. Consistent with a previous report, the treatment of HeLa cells with shHCF-1 or shCDC42 led to chromosome misalignment (Fig. 4b, c) and multinucleation defects (Fig. 4d, e). However, upon co-expression with CDC42F28L, HeLa cells displayed less chromosome misalignment and multinucleation (Fig. 4f, i), indicating that overexpressing activated CDC42 can effectively rescue the mitotic defects induced by HCF-1 knockdown.

### CDC42 restores the expression of Cyclin A-responsive genes downregulated by the loss of HCF-1

**Fig. 5:**
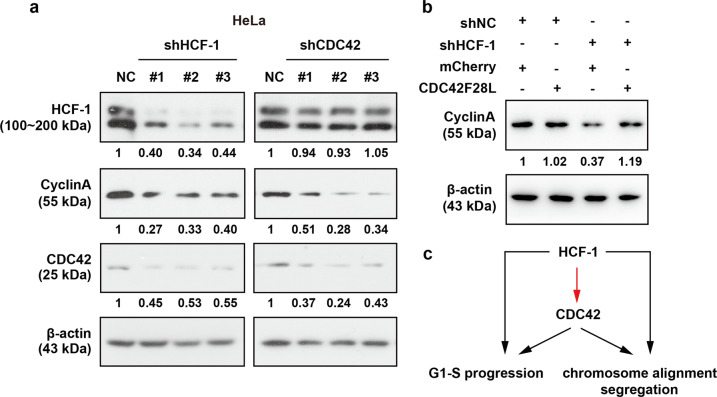
CDC42 overexpression rescued the decreased expression of Cyclin A after HCF-1 knockdown. Immunoblotting analysis of cell lysates after 48 h of transfection. The relative band intensity values were measured with ImageJ software, and the results are shown below the blot. β-actin was used as a loading control. **a** Depletion of HCF-1 and CDC42 induced the downregulation of Cyclin A (*n* = 3). The band intensity values relative to the control values were measured with ImageJ software. **b** Rescue of Cyclin A expression by CDC42F28L overexpression (*n* = 4). **c** HCF-1 coordinates proper cell cycle progression by regulating the expression of CDC42.

Cyclin A is widely accepted as a molecule that is involved in the cell cycle. Its expression changes as the cell transitions to different cell phases, from G0/G1 to S and then to G2/M^25^. We therefore examined whether the expression of Cyclin A is downregulated in HeLa cells treated with either shHCF-1 or shCDC42. Immunoblotting analysis revealed that the expression of Cyclin A was decreased after shHCF-1 or shCDC42 treatment (Figs. 5a and S1c). Since CDC42F28L overexpression led to suppression of cell proliferation in HeLa cells in the absence of HCF-1, we tested whether functional reversion was accompanied by upregulated expression of Cyclin A. As shown in Fig. 5b, HeLa cells that were cotransfected with shHCF-1 and CDC42F28L rescued the protein expression of Cyclin A, which were compared with control.

We confirmed all of these phenotypes in HeLa cells by transfecting shHCF-1-#1 or shCDC42-#2 as well as pCAG-mCherry-CDC42F28L for rescue, and we gained similar conclusions (Figs. S5 and S6). These results indicate that the regulation of CDC42 expression by HCF-1 is correlated with cell cycle progression.

## Discussion

### CDC42 is the transcriptional target of HCF-1

In this study, we found that HCF-1 regulates *CDC42* by targeting the promoter directly. HCF-1 was originally identified to play a key role in lytic infection by herpes simplex virus through the formation of a multi-protein enhancer complex with the viral regulatory protein VP16 and the POU homeodomain transcription factor Oct-1 to direct the transcription of viral immediate early genes^6,26^. Evidence has demonstrated that HCF-1 functions as a mammalian transcriptional cofactor for many transcription regulators, such as Krox20, E2Fs, BAP1 and THAP1^16,27–29^. We need to point out that, although ChIP-seq revealed a series of potential HCF-1 target genes, CDC42 was not functionally validated.

HCF-1 knockdown decreases the expression of CDC42 in HeLa cells and 293ET cells. Furthermore, the expression of CDC42 is altered by HCF-1 during different phases of the cell cycle. These results provide evidence that *CDC42* is the downstream target of HCF-1 for transcriptional regulation. It is reasonable that HCF-1, as a transcription cofactor, functionally interacts with other transcription factors to form a complex, binding to the promoter to activate *CDC42* transcription. The components need to be identified in further studies. Our findings that CDC42 expression is regulated by HCF-1 in the cell cycle will shed light on the study of Rho GTPase expression regulation.

### HCF-1 coordinates phase progression and chromosome segregation by regulating the expression of CDC42

Accumulating evidence has demonstrated the function of HCF-1 as a cell cycle regulator in addition to its function as a transcriptional cofactor. The role of HCF-1 in cell proliferation and mitosis was originally identified in studies of the temperature-sensitive hamster tsBN67 cell line in which a proline-to-serine missense mutation at position 134 (P134S) in the HCF-1N kelch domain induces temperature-sensitive G1/G0 phase cell proliferation arrest and multinucleation defects^1,2^. To gain further insight into the function of HCF-1, investigators have taken advantage of HCF-1 siRNA and have used siRNA-resistant HCF-1 molecules^4^. Through this approach, HCF-1 has been shown to promote G1 phase progression and ensure proper chromosome alignment and segregation^4,5^. The loss of HCF-1 induces a PR-Set7-dependent switch from mitotic H4-K20 monomethylation to dimethylation that contributes to mitotic defects. However, the overexpression of PR-Set7 in the presence of endogenous HCF-1 in HeLa cells is not sufficient to induce improper mitotic defects, suggesting that HCF-1 performs roles in addition to regulating PR-Set7 levels to promote proper cell division^4^.

CDC42 has been shown to be a potential stimulator of E2F^30^, while the Rb-related p107 protein, which is involved in the recruitment of HDAC1 complexes to promoters by HCF-1, inhibits E2F function through the Cyclin A/cdk2 complex^31^. These findings have established an indirect relationship between HCF-1 and CDC42. Our current findings indicate that HCF-1 regulates the expression of CDC42 to promote proper G1 phase progression and cell division, as the overexpression of CDC42 partially rescues G1 delay and multinucleation defects upon the loss of HCF-1. The partial reversal of cell cycle defects by the overexpression of CDC42 in the absence of HCF-1 suggests the possibility that HCF-1 regulates other downstream targets. The overexpression of CDC42 also rescues G1 arrest and the expression of Cyclin A in HCF-1-depleted cells. These findings provide a clue that the function of HCF-1 and CDC42 could have some correlation with HDAC1 complex.

Taken together, we provided evidence that HCF-1 coordinates proper cell cycle progression by regulating the expression of CDC42. Our studies found that HCF-1 and CDC42 depletion induces chromosome misalignment and that multinucleation may be the potential mechanism. These findings deepen our understanding of the cell cycle and may provide a reference to promote the clinical transformation of HCF-1 and improve the clinical diagnosis and treatment of tumours.

## Materials and methods

### Cell culture and synchronization

HeLa cells and 293ET cells were harvested in Dulbecco’s modified Eagle’s medium (DMEM) with 10% foetal bovine serum (FBS). Cells were seeded at a density of 1 × 10^6^ in 10-cm dishes. Synchronization of the cell cycle was achieved by double-thymidine block as follows. HeLa cells in the exponential growth phase were exposed to 2 mM thymidine in DMEM containing 10% FBS for 15 h and then incubated in fresh medium for 9 h. Cells were once again exposed to 2 mM thymidine for 15 h and were cultured in fresh DMEM containing 10% FBS. 2, 4 and 8 h after release, cells were harvested and subjected to analysis. After 6 h, nocodazole was added at a final concentration of 40 ng/ml, and cells were cultured for another 6 h to obtain cells in the M phase. Round mitotic cells were further purified by the shake-off procedure. The collected cells were suspended in fresh DMEM containing 10% FBS to stop the nocodazole-induced arrest.

### Flow cytometry analysis

HeLa cells were harvested after synchronization by double-thymidine block, washed cells twice with cold phosphate buffer saline (PBS), then resuspended in PBS at a concentration of ~1 × 10^6^ cells/ml. A total of 200 µl of the solution was transferred to 4 ml cold 70% ethanol and incubated at −20 °C overnight for cell fixation. Cells were resuspended in PBS, propidium iodide (PI, Sigma–Aldrich) was added to a final concentration of 50 µg/ml, RNase was added to a final concentration of 50 µg/ml, and then cells were incubated for 30 min at 37 °C in the dark. Cells were analysed by flow cytometry (BD Accuri C6, BD Biosciences, USA), and the data were analysed by ModFit LT.

### Plasmid transfection

Human *HCF-1*/*CDC42* shRNA plasmids were constructed based on pLentilox 3.7. The sequences are as follows: shHCF-1-1, 5’-GGG ACA UUC CCA UCA CUU ACG-3'^21^; shHCF-1-2, 5’-GCU UGU CUC AAC CUG GAU ACC-3'^15^; shHCF-1-3, 5’-GCA GUG CUC UGA UUU CCA AUC-3^15^; shCDC42-1, 5’-GGG CAA GAG GAU UAU GAC A-3'^32^; shCDC42-2, 5’-UGA GAU AAC UCA CCA CUG U-3'^33^ and shCDC42-3, 5’-CAG UUA UGA UUG GUG GAG A-3'^33^. A nontargeting shRNA plasmid (5’-UUC UCC GAA CGU GUC ACG U-3’) was used as a nonspecific control. DNA (1.6 µg) was used for transfection in 12-well cell culture plates, while 0.8 µg was used for transfection in 24-well cell culture plates. The transfections were performed 48 h before each cell harvest. pCAG-mCherry and pCAG-mCherry-CDC42F28L plasmids were constructed for overexpression experiments. All plasmids were extracted by a TIANprep Mini Plasmid Kit (TIANGEN, DP103). Endotoxin removal reagent was provided by Solarbio (E1040). All transfections were carried out by Lipofectamine 2000 according to the manufacturer’s instructions (Invitrogen, 11668).

### Quantitative real-time RT-PCR

Quantitative RT-PCR was carried out using SYBR Premix Ex Taq Master Mix and a 2-Step kit (TaKaRa, Dalian, China). Total cellular RNA was isolated using TRIzol reagent according to the manufacturer’s instructions (Life Technologies). Total RNA (2.0 μg) was used for cDNA synthesis using oligo dT primers (Transgene, Beijing, China), and 1/10 of the cDNA volume was used for quantitative PCR. PCR amplification was carried out using a CFX96 Touch System (BioRad, Hercules, CA, USA). The PCR conditions were carried out according to the manufacturer’s instructions. The Ct values were normalized to that of the human glyceraldehyde-3-phosphate dehydrogenase (GAPDH) gene. The ΔΔCt method was used to determine the relative expression level of the target genes. All samples were run in triplicate in each experiment. Each assay was repeated three times. All primers were synthesized by TSINGKE (Beijing, China). The primers were as follows: for *HCF-1*, 5’ - GGC AGT GCT CTG ATT TCC A -3’ and 5’ - TTC AGG ATT GTT CCC GCT-3’; for *CDC42*, 5’ - GGA TTA TGA CAG ATT ACG ACC G-3' and 5’ - GTT ATC TCA GGC ACC CAC TTT-3’; and for *GAPDH*, 5’ - AAA TTG AGC CCG CAG CCT-3' and 5’ - CCC AAT ACG ACC AAA TCC GT-3’.

### Semi-quantitative RT-PCR

Total cellular RNA was extracted with TRIzol reagent and reverse-transcribed with MMLV and oligo dT primer (New England Biolabs). *HCF-1* cDNA was amplified with the following primers: 5’-AGC TGA AGA AGC AGG AGC TG-3’ and 5’-GGC TTG GTG GTG TAG TCG AT-3’. *CDC42* cDNA was amplified with the following primers: 5’-ACG ACC GCT GAG TTA TCC AC-3’ and 5’-GCC AGC TTT TCA GCA GTC TC-3’. *GAPDH* cDNA was amplified with the following primers: 5’-ACC ACA GTC CAT GCC ATC AC-3’ and 5’-TCC ACC ACC CTG TTG CTG TA-3’. Real-time PCR reagents were provided by TAKARA (TaKaRa, RR420A). Reactions and analyses were performed using a Bio-Rad CFX Connect Real-Time PCR System.

### Immunoblotting analysis

Whole cells were lysed in TNTE buffer (50 mM Tris-HCl, pH 7.4, 150 mM NaCl, 1 mM EDTA, pH 8.0, 1% Triton X-100, 25 mM NaF, 1 mM Na_2_VO_3,_ and 100 μg/mL DTT) containing protease and phosphate inhibitors. Cells lysates were subjected to electrophoresis on SDS polyacrylamide gels, and the proteins were transferred to nitrocellulose membranes (Pall Corp). The blots were blocked with 5% skim milk, followed by incubation with an anti-HCF-1 antibody (Bethyl Laboratories, A301-399A, diluted 1:2000), an anti-CDC42 antibody (Santa Cruz, sc-87, discontinued antibody or sc-8401, diluted 1:200), an anti-RhoA antibody (Abcam, ab187027, diluted 1:1000), an anti-Rac1 antibody (Abcam, ab33186, diluted 1:1000), an anti-E2F-1 antibody (Santa Cruz, sc-251, diluted 1:300), an anti-Cyclin A antibody (Santa Cruz, sc-239, diluted 1:300), an anti-Cyclin D antibody (Abcam, ab16663, diluted 1:1000), an anti-Cyclin E antibody (Santa Cruz, sc-377100, diluted 1:300), an anti-p27KIP antibody (Santa Cruz, sc-1641, diluted 1:300) or an anti-β-actin antibody (Sigma, A5441, diluted 1:5000). The blots were then incubated with secondary antibody conjugated to horseradish peroxidase (Amersham Biosciences) and visualized by a chemiluminescent detection system (Amersham Biosciences).

### Chromatin immunoprecipitation (ChIP) assay

Approximately 2 × 10^7^ subconfluent HeLa cells grown in DMEM medium were collected, and native protein-DNA complexes were cross-linked by treatment with 1% formaldehyde for 10 min. Chromatin sonication was performed by an ultrasonic cell disruptor (JY92-II, NingBo Scientz Biotechnology Co., Ltd.). The ChIP assay was generally carried out with the MAGnify Chromatin Immunoprecipitation System (Applied Biosystems™, 492024). Briefly, equal aliquots of isolated chromatin were subjected to immunoprecipitation with 5 µg anti-HCF-1 antibody or normal IgG from the same species as the control. The DNA fragments associated with specific immunoprecipitates or with negative control IgG were isolated and used as templates for subsequent PCRs to amplify the *CDC42* promoter sequences. The primers were as follows: for the upstream region (−1362 to −862), 5’ -ATC TCC TGA CCT CGT GAT CCG-3’ and 5’ -TCA CTG CAA TCT CGA CGT CC-3’; for the -881 to -575 region, 5’ -GGA CGT CGA GAT TGC AGT GA-3’ and 5’ -TCT GAA AGG GCT GGT GAC TT-3’; and for the -594 to -111 region, 5’ -AGT CAC CAG CCC TTT CAG A-3' and 5’ -GGG CTA TGC TCT GCA TGT TT-3’.

### DNA pull-down assay

DNA pull-down assays were carried out following a previous description^34^. HeLa cells were lysed by sonication in HKMG buffer (10 mM HEPES, pH 7.9, 100 mM KCl, 5 mM MgCl_2_, 10% glycerol, 1 mM DTT and 0.5% NP-40) containing protease and phosphate inhibitors. Cellular debris was removed by centrifugation. Two milligrams of cell extract was precleared with 40 μl of streptavidin-agarose beads (Thermo Scientific, 20347) for 1 h at 4 °C and then incubated with 2 μg of biotinylated double-stranded oligonucleotides and 40 μg of LightShift Poly(dI-dC) (Thermo Scientific, 20148E) for 16 h at 4 °C. DNA-bound proteins were collected with 60 μL of streptavidin-Sepharose beads for 1 h at 4 °C, washed twice with HKMG buffer, separated by SDS-PAGE, and identified by western blot. The biotinylated double-stranded oligonucleotides were amplified using 5’ terminal biotinylated primers with the same sequences used for ChIP detection.

### Immunofluorescence assay

HeLa cells on coverslips were transfected with shRNA-producing plasmids, pCAG-mCherry-CDC42F28L or their controls for 48 h. After fixation with 4% paraformaldehyde for 30 min, cells were permeabilized with 0.3% Triton X-100 in PBS for 15 min and then blocked for 30 min with 3% bovine serum albumin (BSA) at room temperature. Incubation with antibodies against β-tubulin (Sigma–Aldrich, T4026) was performed, followed by incubation with an Alexa Fluor^®^ 647-conjugated secondary antibody (Invitrogen™). Nuclei were counter-stained with DAPI (Sigma–Aldrich). Confocal fluorescence images were obtained with a Olympus FV-1000 system.

### EdU incorporation assay

Transfected cells grown on coverslips were incubated with 10 nM EdU (5-ethynyl-2’-deoxyuridine) 1 h before fixation with 4% paraformaldehyde at 4 °C. EdU incorporation was determined by staining using a Click-iT™ Plus EdU Alexa Fluor™ 647 Imaging Kit (Invitrogen™, C10640). Cells were also counter-stained with DAPI. Confocal fluorescence images were obtained with an Olympus FV-1000 system.

## Supplementary information

Supplementary Figure Legends

Figure S1

Figure S2

Figure S3

Figure S4

Figure S5

Figure S6
