# Supplementary material for: HCF-1 promotes cell cycle progression by regulating the expression of CDC42
Source: Cell Death Dis. 2020 Oct 23;11(10):907. doi: 10.1038/s41419-020-03094-5 (PMC7584624; doi:10.1038/s41419-020-03094-5)
Supplement: Supplementary file 1 — Supplementary Figure Legends [file 41419_2020_3094_MOESM1_ESM.docx]

**HCF-1 promotes cell cycle progression by regulating the expression of CDC42**

**Pan Xiang ^1,3^, Fei Li^1,3^, Zhihua Ma^1^, Jiping Yue^1^, Cailing Lu^1^, Yuangang You^1^, Lin Hou^1^, Bin Yin^1^, Boqin Qiang^1^, Pengcheng Shu^1*^, Xiaozhong Peng^1,2*^**

^1^State Key Laboratory of Medical Molecular Biology, Department of Molecular Biology and Biochemistry, Institute of Basic Medical Sciences, Medical Primate Research Center, Neuroscience Center, Chinese Academy of Medical Sciences, School of Basic Medicine Peking Union Medical College, Beijing, China

^2^Institute of Medical Biology, Chinese Academy of Medical Sciences, Peking Union Medical College, Kunming, China

^3^These authors contributed equally to this work.

* To whom correspondence should be addressed.

Email: [pengxiaozhong@pumc.edu.cn; pengcheng_shu@ibms.pumc.edu.cn](mailto:pengxiaozhong@pumc.edu.cn;%20pengcheng_shu@ibms.pumc.edu.cn)

**Supplemental Information**

(Includes 6 figures)

**Fig. S1. the status of GTPase and G1-S cyclins upon decreasing HCF-1/CDC42 protein levels**

(a) Total protein from HeLa cells treated with HCF-1 targeting plasmids or non-targeting plasmids (abbreviated NC or shNC) were extracted after 48 h of transfection and then analysed by immunoblotting for HCF-1, CDC42, RhoA and Rac1 expression (n=3). Depletion of HCF-1 led to decrease CDC42 expression in HeLa cells. Both Rac1 and RhoA were not altered upon HCF-1 knock down. (b) Western blot showing CDC42 protein levels. These cells were treated with HCF-1/CDC42-targeting plasmids or non-targeting plasmids and then synchronized and incubated with EdU (n=3). (c) The protein levels of HCF-1, CDC42, Cyclin A, Cyclin E, Cyclin D and p27KIP upon HCF-1 knock down. β-Actin was used as a control (n=3).

**Fig.S2. Standard curve of DNA concentration**

Genomic DNA from HeLa cells was serially diluted to generate standard products to establish a standard curve of the relative threshold circulation number (Ct). The X axis represents the logarithm (base 10) of the DNA concentration (ng/ul).

**Fig.S3. mCherry CDC42 fusion overexpression in HeLa cells**

Total Protein of HeLa cells treated with CDC42-fusion protein plasmids or control plasmid were extracted after 48h of transfection and then analyzed by immunoblotting for CDC42-fusion protein expression. GAPDH were used as a control (n=4).

**Fig.S4. Cell synchronization was monitored by flow cytometry**

Cells were harvested at different time points (0, 2, 4, 8 h) after double thymidine block, and the nocodazole-treated cell pool were also harvested. The synchronizing degree of cells was monitored by flow cytometry.

**Fig.S5. HCF-1 promotes G1 progression by regulating CDC42 expression**

HCF-1 and CDC42 depletion induced cellular phenotypes in HeLa cells. (a) HCF-1 and CDC42 depletion induced G1 arrest in HeLa cells. Cells were transfected with the shRNA-producing plasmid. GFP was expressed in successfully transfected cells. Scale bar: 50 µm. (b) Quantification of EdU incorporation after HCF-1 and CDC42 knockdown. The ratio of EdU incorporation was 73.02%, 44.51% and 49.14% in transfected groups, respectively (n=6). (c) Cells were transfected with the shHCF-1 and CDC42F28L vectors. Both GFP and mCherry were expressed in successfully transfected cells. Scale bar: 50 µm. (d) Quantification of EdU incorporation in the co-expression cells. The ratio of EdU incorporation was 84.59%, 45.5%, 79.65% and 80.74% in transfected groups, respectively (n=8). Results are expressed as mean ± SD. (****p < 0.001*; NS: no significant difference. Student's t-test).

**Fig.S6. HCF-1 promotes** **mitosis by regulating CDC42 expression**

HCF-1 and CDC42 depletion induced chromosome misalignment and multinucleation in HeLa cells. (a, c) Cells were transfected with shRNA-producing plasmids. GFP was expressed in successfully transfected cells. Cells were counter-stained with β-tubulin antibody. Scale bar: 10 µm. (b) Quantification of chromosome misalignment after HCF-1 and CDC42 knockdown. The ratio of chromosome misalignment was 5.6%, 40.4% and 34.5% in transfected groups, respectively (n=15). (d) Quantification of multinucleation after HCF-1 and CDC42 knockdown. The ratio of chromosome misalignment was 1%, 9.4% and 12% in transfected groups, respectively (n=10). (e, g) Cells were transfected with shHCF-1 and CDC42F28L vectors. Both GFP and mCherry were expressed in successfully transfected cells. Cells were counter-stained with β-tubulin antibody. Scale bar: 10 µm. (f) Quantification of chromosome misalignment in the co-expression cells. The ratio of chromosome misalignment was 4.3%, 39.9%, 2% and 2% in transfected groups, respectively (n=15). (h) Quantification of multinucleation in the co-expression cells. The ratio of chromosome misalignment was 3.7%, 15.7%, 2.3% and 2.5% in transfected groups, respectively (n=10). Results are expressed as mean ± SD. (**p < 0.05, **p < 0.01, ***p < 0.001*. Student’s t-test).
