# Supplementary figures and images for: HCF-1 promotes cell cycle progression by regulating the expression of CDC42
Source: Cell Death Dis. 2020 Oct 23;11(10):907. doi: 10.1038/s41419-020-03094-5 (PMC7584624; doi:10.1038/s41419-020-03094-5)

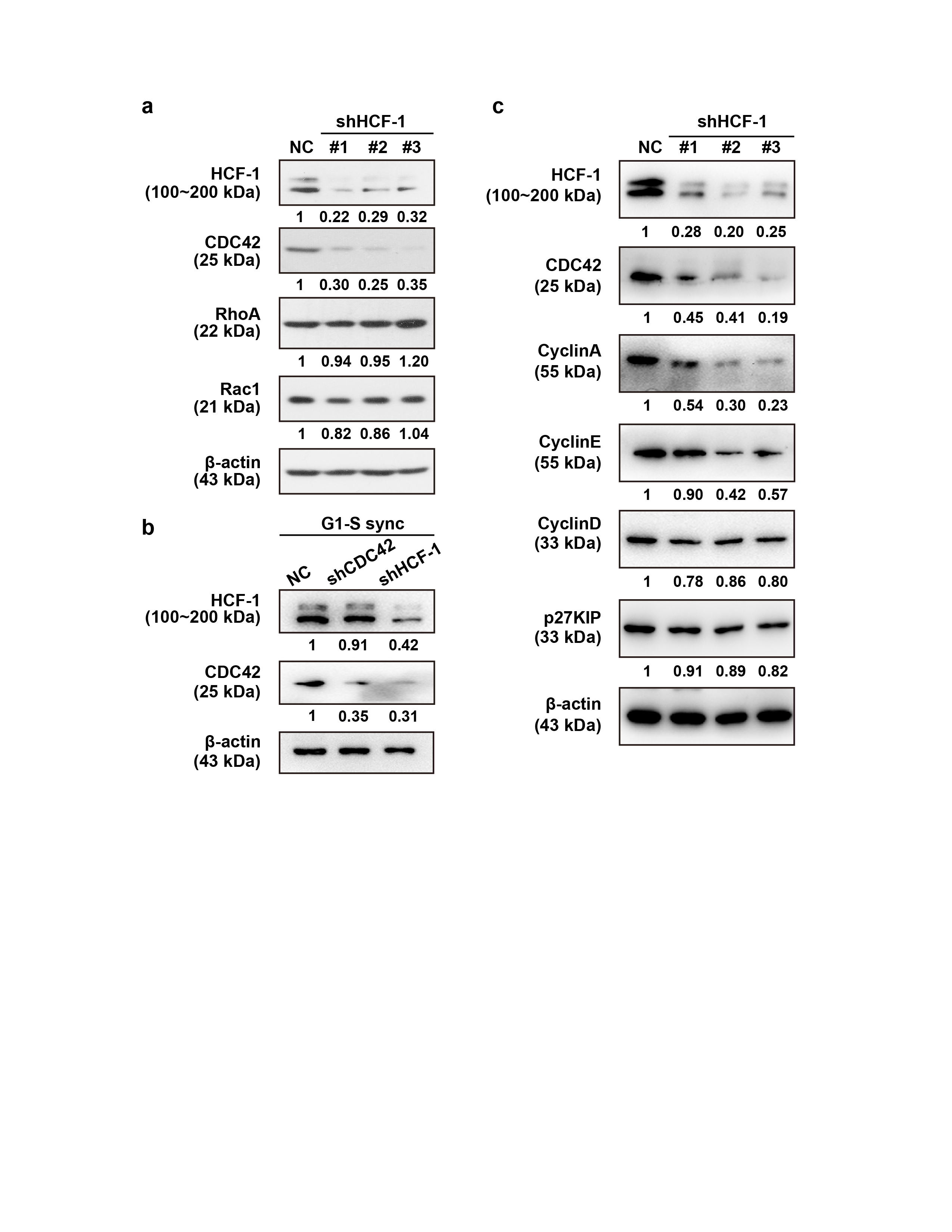

Supplement: Supplementary file 2 — Figure S1 [file 41419_2020_3094_MOESM2_ESM.png]

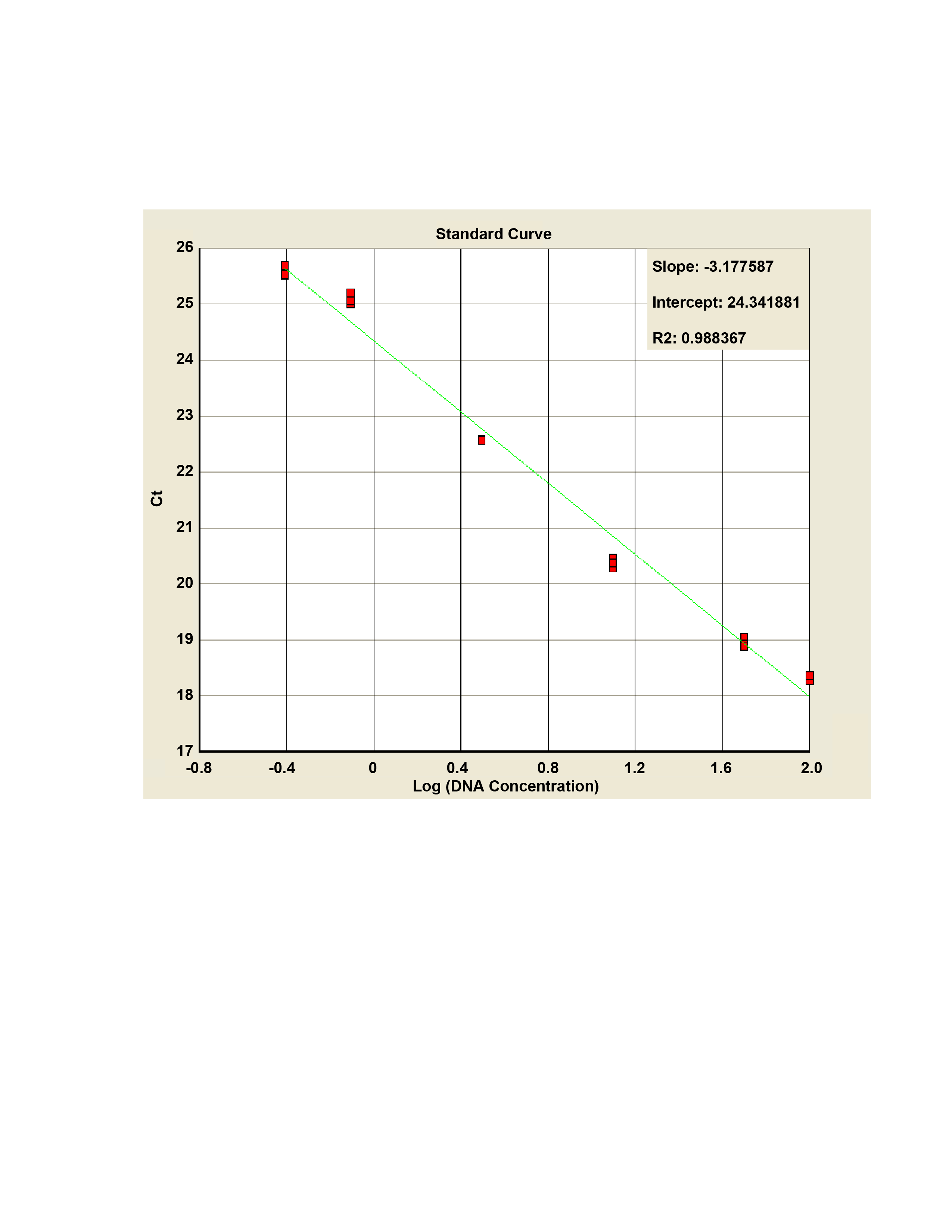

Supplement: Supplementary file 3 — Figure S2 [file 41419_2020_3094_MOESM3_ESM.png]

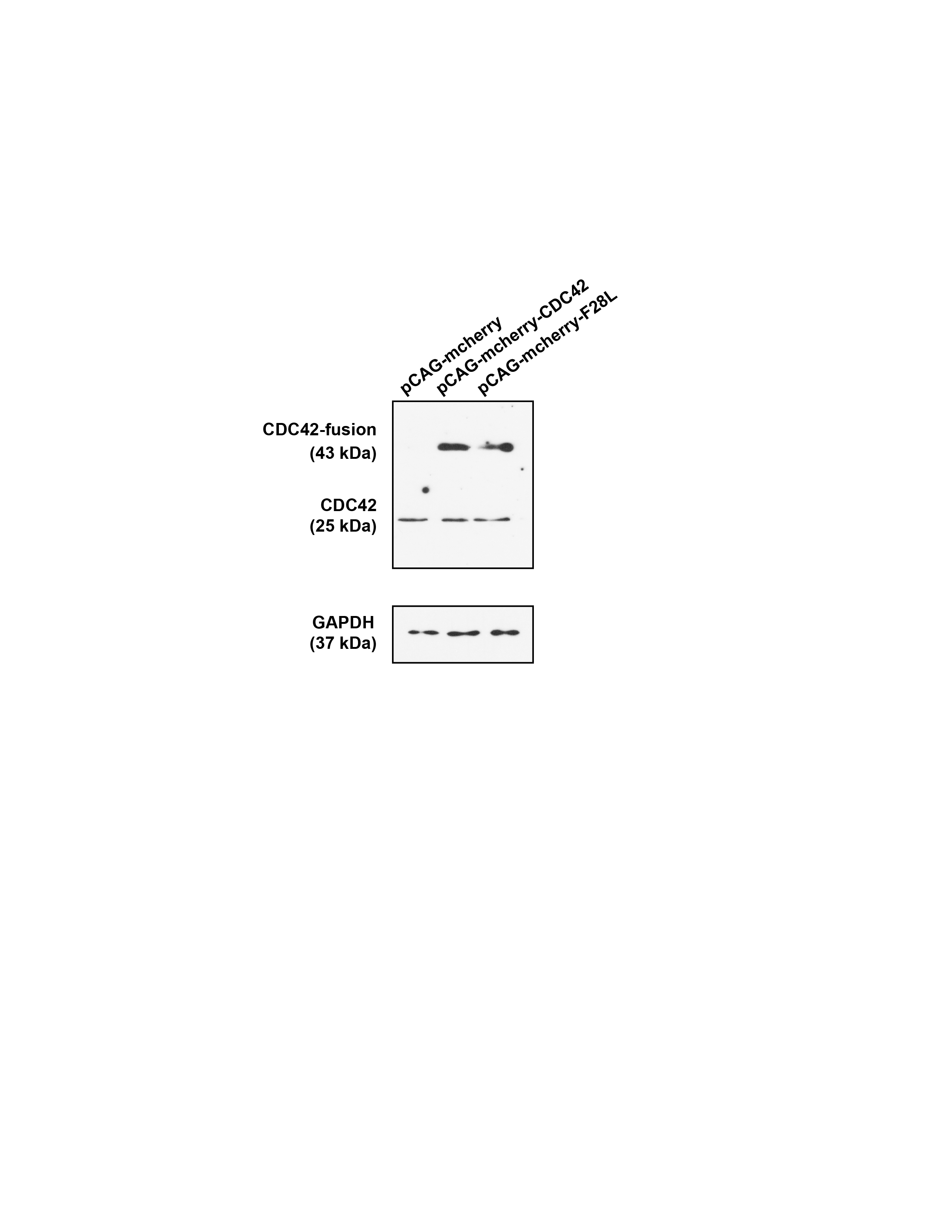

Supplement: Supplementary file 4 — Figure S3 [file 41419_2020_3094_MOESM4_ESM.png]

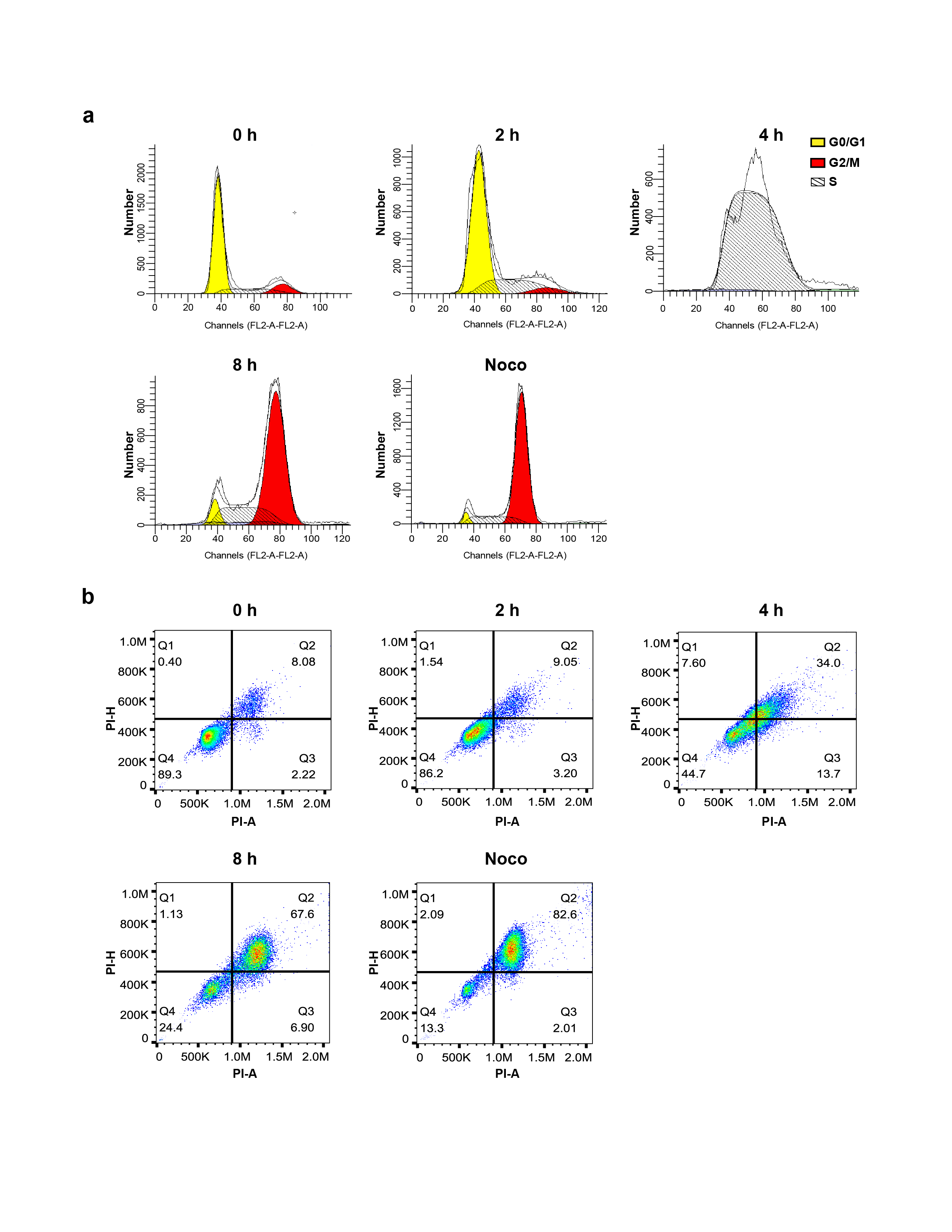

Supplement: Supplementary file 5 — Figure S4 [file 41419_2020_3094_MOESM5_ESM.png]

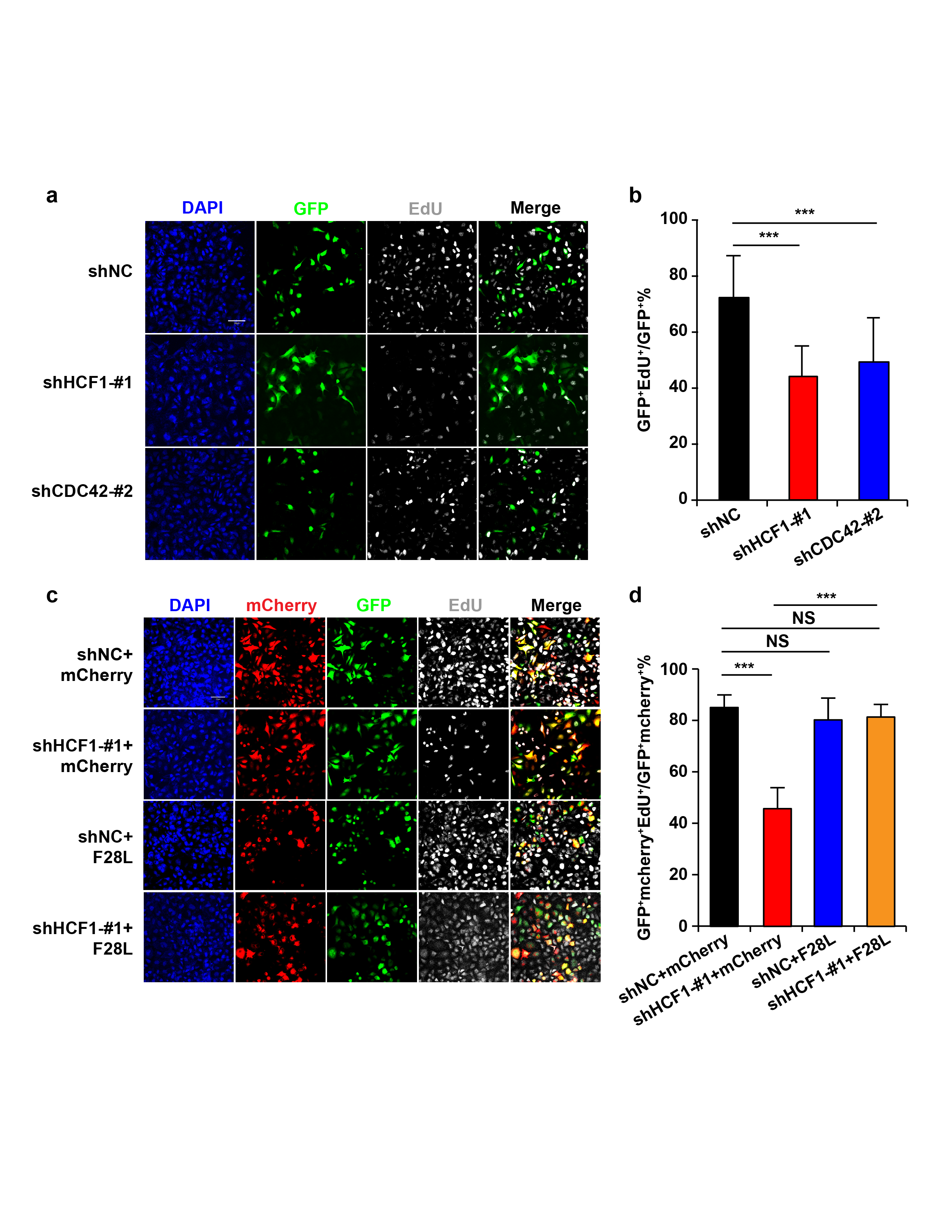

Supplement: Supplementary file 6 — Figure S5 [file 41419_2020_3094_MOESM6_ESM.png]

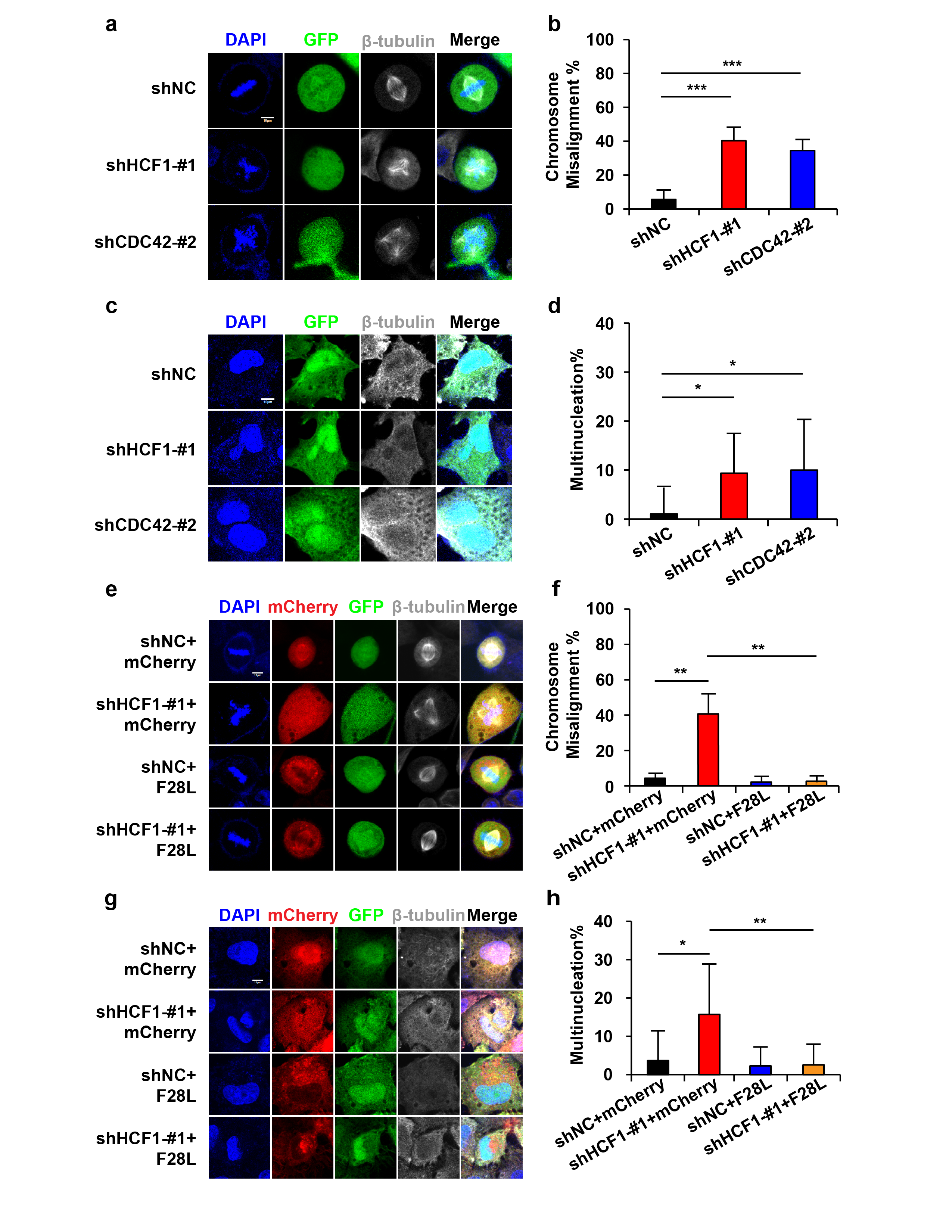

Supplement: Supplementary file 7 — Figure S6 [file 41419_2020_3094_MOESM7_ESM.png]
